# Active Accumulation of Spherical Analytes on Plasmonic Hot Spots of Double-Bent Au Strip Arrays by Multiple Dip-Coating

**DOI:** 10.3390/nano9050660

**Published:** 2019-04-26

**Authors:** Jinhyung Lee, Eun-Ah You, Do Won Hwang, Shinill Kang, Jung-Sub Wi

**Affiliations:** 1 Center for Nano-Bio Measurement, Korea Research Institute of Standards and Science, Daejeon 34113, Korea; jinhyung@kriss.re.kr (J.L.); eayou@kriss.re.kr (E.-A.Y.); 2 School of Mechanical Engineering, Yonsei University, Seoul 03722, Korea; 3 Department of Nuclear Medicine, Seoul National University Hospital, Seoul 03080, Korea; hdw6592@snu.ac.kr

**Keywords:** localized surface plasmon resonance, dip-coating, capillary force, exosome

## Abstract

To achieve sensitive plasmonic biosensors, it is essential to develop an efficient method for concentrating analytes in hot spots, as well as to develop plasmonic nanostructures for concentrating light. In this study, target analytes were delivered to the surface of double-bent Au strip arrays by a multiple dip-coating method; they were self-aligned in the valleys between neighboring Au strips by capillary forces. As the valleys not only accommodate target analytes but also host strong electromagnetic fields due to the interaction between adjacent strips, sensitive measurement of target analytes was possible by monitoring changes in the wavelength of a localized surface plasmon resonance. Using the proposed plasmonic sensor and target delivery method, the adsorption and saturation of polystyrene beads 100 nm in size on the sensor surface were monitored by the shift of the resonance wavelength. In addition, the pH-dependent stability of exosomes accumulated on the sensor surface was successfully monitored by changing the pH from 7.4 to 4.0.

## 1. Introduction

Biosensors based on localized surface plasmon resonance (LSPR), the collective oscillation of electrons in nanostructured noble metals induced by resonant light, have been extensively investigated owing to their molecular-level sensitivity, rapid and label-free detection, and simple instrumentation [1,2,3,4]. Since LSPR sensors monitor the resonance wavelength shifts caused by analytes adsorbed on the metal surface, fabrication of uniform plasmonic nanostructures with sharp and clean resonance spectra is a prerequisite for achieving sensitive and reliable LSPR sensors [1,2,3,4,5,6,7]. Once well-defined plasmonic nanostructures are obtained, the delivery of analytes to plasmonic hot spots is essential to fully utilize the sensor performance. However, despite the availability of a number of reports on the fabrication of various metallic nanostructures and their application in plasmonic sensors, only a few studies have focused on the locating of analytes on sensing spots [8,9,10].

In this report, we present an efficient method for locating analytes in plasmonic hot spots. By a template-assisted self-assembly during the dip-coating process [11,12,13,14], target analytes spontaneously align in the valleys between neighboring Au strips, where electromagnetic fields are locally enhanced. During repeated dip-coating, analytes accumulate at the hot spots, and this phenomenon is monitored for changes in LSPR wavelength. Furthermore, we demonstrate that the proposed method can be used to monitor the stability of exosomes, extracellular vesicles (<100 nm in size), attracting much scientific and engineering interest as biomarkers for diagnosing diseases and as delivery vehicles for bioresorbable drugs [15,16,17,18].

## 2. Experimental Methods

### 2.1. Nanofabrication of Double-Bent Au Strip Arrays

**Figure 1:**
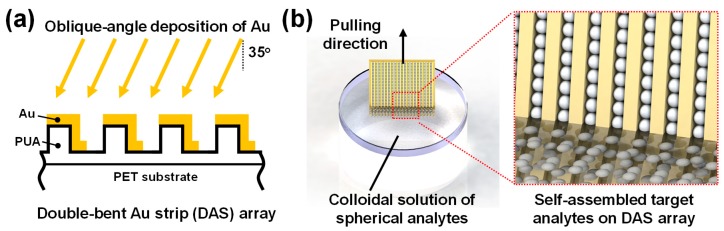
Schematic illustration of sample preparation. (**a**) Double-bent Au strips (DAS) were thermally deposited at an oblique angle on UV-imprinted polymer nanograting structures. PUA and PET are polyurethane acrylate and polyethylene terephthalate, respectively. (**b**) A dip-coating process was used to assemble polystyrene beads or exosomes on the DAS sensors; the dip-coating process was repeated to accumulate analytes.

The sample preparation process is illustrated in Figure 1. Initially, one-dimensional grating patterns with a period of 200 nm (1:1 line/space) and a height of 100 nm were generated on polyurethane acrylate (PUA)-coated polyethylene terephthalate (PET) substrate by UV-nanoimprint lithography. Then, a 30 nm-thick Au film was thermally evaporated at an oblique angle to the polymer nanograting. Because of the tilted deposition angle (35° from the surface normal direction) and a shadowing effect of the line grating, a double-bent Au strip (DAS) array was spontaneously formed in two steps, namely, UV-nanoimprint lithography and Au deposition. More details on the fabrication of DAS arrays can be found in our previous report, in which the refractive index sensitivity of about 210 nm refractive index unit (RIU)^−1^ and a figure of merit (FOM) of 4.2 for DAS arrays were demonstrated [19].

### 2.2. Active Accumulation of Spherical Analytes on DAS Arrays by Multiple Dip-Coating

After preparation of DAS arrays, the samples were vertically dipped in a colloidal solution of polystyrene (PS) beads or exosomes and pulled out at a speed of 0.01 mm/s. A 1 wt % aqueous solution of PS beads was purchased from ThermoFisher Scientific (Nanosphere^TM^ size standard 3100A, Waltham, MA, USA). Between each coating cycle in the multiple dip-coating process, the samples were dried for 5 min in ambient atmosphere and then immersed again. After the dip-coating process was completed, the absorbance curves were measured using a UV-Vis spectrophotometer (UV-2600, Shimadzu, Kyoto, Japan) and the surfaces were observed using scanning electron microscopy (SEM, S-4800 FE-SEM, Hitachi, Tokyo, Japan).

### 2.3. Assessment of Exosome Stability

Exosomes were isolated from human fibroblast cells grown in Dulbecco’s minimal essential medium supplemented with 10 % fetal bovine serum, 100 U/mL penicillin/streptomycin at 37 °C. The supernatant was centrifuged at 800× *g* for 5 min and 2000× *g* for 10 min to remove cellular debris. The ultracentrifugation step was conducted in cell debris-removed supernatant at 100,000× *g* for 3 h to collect the pellet (Beckman, Brea, CA, USA). The exosomal protein concentration of 1 μg/μL was measured using a Pierce BCA Protein Assay Kit. To examine the pH-dependent stability of exosomes, the exosome-adsorbed DAS arrays were immersed in a TRIzol reagent (Invitrogen, Waltham, MA, USA), 1× phosphate buffer solution with pH 7.4 (Merck, Darmstadt, Germany), and citric acid/sodium hydroxide buffer solutions with pH 4.0 and 5.0 (Merck, Darmstadt, Germany).

## 3. Results and Discussion

### 3.1. Multiple Dip-Coating of Spherical Analytes on Nanoplasmonic Sensors

**Figure 2:**
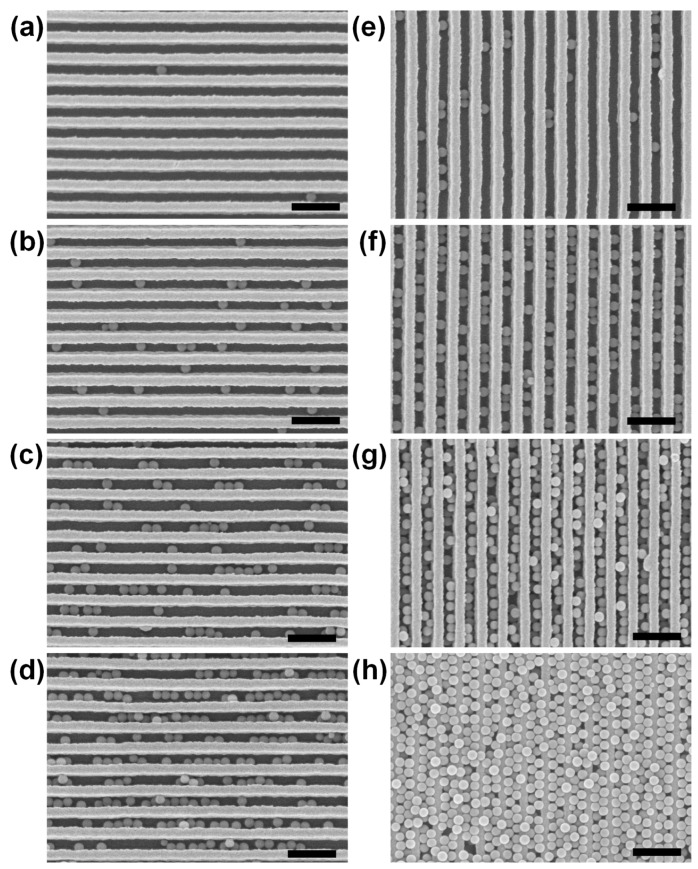
SEM images of the polystyrene (PS) bead-coated sample surfaces according to the direction and number of repeated dip-coating cycles. The dipping and pulling directions of DAS arrays were (**a**–**d**) perpendicular and (**e–h**) parallel to the direction of the length of Au strips. Samples were dip-coated (**a**,**e**) once, (**b**,**f**) three times, (**c**,**g**) five times, and (**d**,**h**) seven times. Scale bars represent 500 nm.

To examine the anisotropic wetting effect of the one-dimensional grating structure [20,21,22], the dipping and pulling directions of the DAS arrays were set parallel or perpendicular to the length direction of the Au strips. Figure 2 shows the SEM images of the PS bead-coated sample surfaces, depending on the direction and number of dip-coating cycles. Due to capillary forces induced on the three-phase (liquid–vapor–substrate) contact line [11,12,13,14], PS beads spontaneously assembled in the valleys between neighboring DAS structures and adsorbed on the surface by the van der Waals forces, regardless of the dip-coating direction. However, it should be noted that the density of the PS beads on the samples where the dip-coating direction was parallel to the Au strip (hereinafter referred to as par-dip sample) was much higher than that on the samples with a perpendicular dip-coating direction (hereinafter referred to as the per-dip sample). For example, in the case of a triple dip-coating, the population of PS beads of the par-dip sample was 154 in the SEM image (Figure 2f), which was approximately five times larger than that of the per-dip sample (Figure 2b). It is believed that this difference was caused by anisotropic wettability and evaporation-induced flow near the contact line. Because the liquid contact angle in the direction parallel to the Au strip was smaller than the contact angle along the perpendicular direction, the par-dip sample with its long meniscus could secure a longer time for positioning the PS beads than the per-dip sample. In addition, the contact line with its long meniscus could induce an outward flow of the PS beads during evaporation; this phenomenon is known as the coffee stain phenomenon [23,24]. Accordingly, deposits of self-assembled beads could accumulate much more on the par-dip sample than on the per-dip sample during the multiple dip-coating process. In the case of the par-dip sample, the valleys between neighboring DAS structures were almost filled with PS beads after five repeated dip-coating cycles, as shown in Figure 2g.

**Figure 3:**
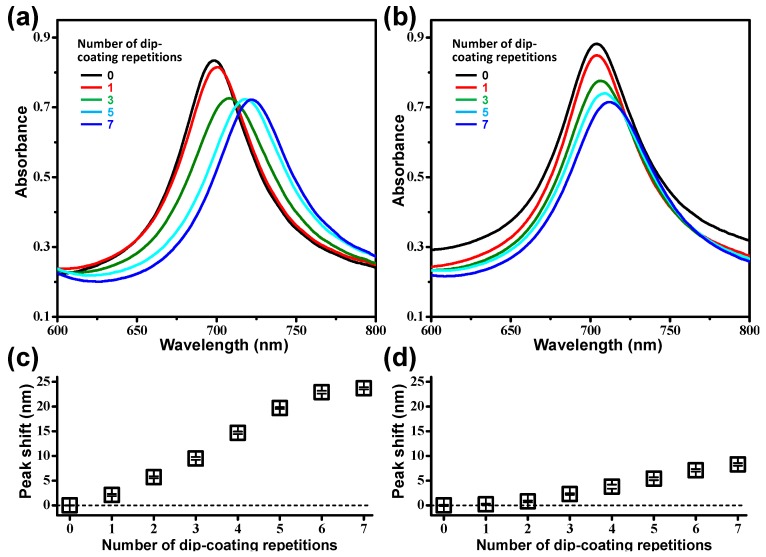
(**a**,**b**) Absorbance curves and (**c**,**d**) localized surface plasmon resonance (LSPR) peak shifts of PS bead-coated DAS arrays as functions of the number of dip-coating repetition cycles. The data in Figure 3a,c and Figure 3b,d were obtained from the par-dip and per-dip samples, respectively. All the data in Figure 3c,d showed statistically significant differences (P-value < 0.05), except for the first two data in Figure 3d (single and double dip coatings).

The valleys between the Au strips not only accommodate PS beads by capillary forces, but also act as hosts to strong plasmonic fields due to the interactions between adjacent strips, as was demonstrated in experimental and simulation studies [25]. Therefore, the PS beads self-assembled in the valley between the DAS arrays can induce a change in the resonance conditions of the Au strips, resulting in a spectral shift of the LSPR wavelength. Figure 3 shows the LSPR shifts of the per- and par-dip samples depending on the number of dip-coating repetitions. The LSPR peak shifts of the par-dip samples were much larger than those of the per-dip samples due to a greater number of dense bead deposits on the sample surfaces. This observation is in good agreement with the SEM images in Figure 2. As the LSPR peak shift was accompanied by a change in the amplitude of absorbance, as shown in Figure 3a,b, it would be possible to monitor the change in absorbance at a specific wavelength (e.g., the initial resonant wavelength) as an alternative signal reading method.

Figure 3a,c also indicates that the peak shift generated by the dip-coating was reduced after five repetitions of the dip-coating process. Once the valleys were filled with PS beads, the additional beads adsorbed on the upper sides of the DAS structures were too far away from the plasmonic fields developed in the valleys, and hence they did not significantly affect the resonant condition of the Au strips. For the same reason, analytes that are too small or beads that are too large compared to the dimensions of the valley are not very sensitive to DAS arrays, as demonstrated by experiments and theoretical calculations in our previous report [25]. Therefore, the dimensions of the DAS structures can be adapted to the size of a particular analyte, and the gold surface of DAS can be functionalized with antibodies for target-specific detection.

### 3.2. Assessment of Exosome Stability by Nanoplasmonic Sensors

In addition to monitoring the adsorption of analytes, it is also possible to monitor desorption or degradation of analytes adsorbed on DAS arrays by measuring the LSPR shifts. Thus, DAS arrays in combination with the proposed multiple dip-coating method were used to assess the stability or degradability of exosomes in neutral or acidic microenvironment known to be associated with their drug delivery efficacy [17,18]. Exosomes dispersed in a phosphate-buffered solution at a concentration of 1 μg/μL were accumulated on DAS arrays by thrice dip-coating. The dip-coated exosomes resulted in an 8 nm red shift of the LSPR peak (Supplementary Materials, Figure S1). For the case when a 10 μL drop of the same exosome sample was dried on a DAS array, the LSPR peak shift was less than 2 nm (Supplementary Materials, Figure S2a).

**Figure 4:**
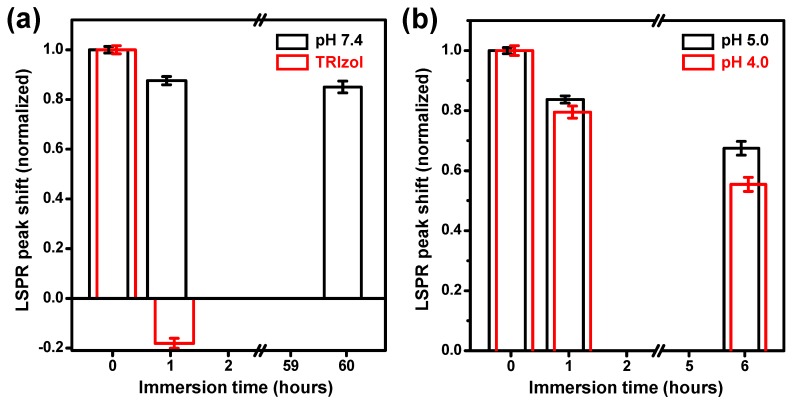
Normalized LSPR peak shifts measured by exosome-coated DAS arrays. After dip-coating thrice with exosomes, the samples were immersed in a TRIzol reagent (red bars in (**a**)) and buffer solutions with pH 7.4 (black bars in (**a**)), 5.0 (black bars in (**b**)), and 4.0 (red bars in (**b**)). The peak shifts were divided by the initial shift values, measured after dip-coating thrice with exosomes for each sample.

To examine the pH-dependent stability of exosomes, the exosome-adsorbed DAS arrays were immersed in a TRIzol reagent and buffer solutions with pH 7.4, 5.0, and 4.0. TRIzol reagent for complete fragmenting of exosomes was used as a control [26]. After treatment with TRIzol reagent, the LSPR peak of the exosome-adsorbed DAS array was blue-shifted (9.5 nm) and, thus, returned to its original position within 1 h (red bars in Figure 4a). The 1.5 nm blue shift from the original position may be the result of damage to the polymer substrate (PET) by the reagent. Compared with TRIzol reagent, it was confirmed that buffer solutions with pH 7.4, 5.0, and 4.0 did not make any noticeable damage on the DAS arrays and the substrate (Supplementary Materials, Figure S2b). In the buffer solution of pH 7.4, the physiological condition was stable for exosomes, and no apparent changes in the LSPR peak were observed, although the exosome-coated DAS array was immersed for up to 60 h (black bars in Figure 4a). Compared to the previous two extremely unstable (TRIzol) and stable (pH 7.4) cases, the peak shifts of the samples immersed in acidic buffer solutions (pH 4.0 and 5.0) exhibited gradual changes, as shown in Figure 4b. The gradual blue shift in the LSPR peaks indicates that the exosomes on the DAS array were partially desorbed from the sample surface or degraded under acidic conditions with an increase in the immersion time. As the desorption of exosomes in the pH 7.4 buffer solution was not very significant, exosome degradation is considered a possible cause of the blue shift of the LSPR peak in acidic buffer solutions. Petelsak et al. demonstrated that a lipid bilayer membrane is subject to greater interfacial tension under acidic conditions than under neutral conditions [27]. Therefore, in pH 4.0 and pH 5.0 buffer solutions, increased interfacial tension of the lipid membrane can reduce the stability of exosomes and result in a blue shift of the LSPR peak. In addition to testing the exosome stability demonstrated in this report, it is possible to monitor the loading and unloading of drug molecules from the exosomes under various physiological conditions that are the subjects of our further studies.

## 4. Conclusions

For effective placement of target analytes on sensor surfaces and their monitoring, we present a straightforward measurement method based on nanoplasmonic sensors of double-bent Au strip arrays, multiple dip-coating of analytes, self-alignment of analytes in the region of strong plasmonic fields, and spectrometric monitoring of LSPR peaks. Using this method, closely packed PS beads in the valleys of the DAS array and pH-dependent stability of the exosomes were successfully monitored in terms of shifts in the LSPR peaks. As a small amount of target analytes can accumulate in the plasmonic hot spot due to multiple dip-coating cycles, and the LSPR peak can be measured with a conventional UV-vis spectrometer under physiological conditions, it is expected that the proposed measurement platform will be useful for studying the stability of various drug delivery vesicles and their efficiencies.

## Supplementary Materials

The following are available online at https://www.mdpi.com/2079-4991/9/5/660/s1, Figure S1: (a) Measured absorbance curves of the DAS array (black) before and (red) after dip-coating thrice with exosomes and (blue) after immersion in a TRIzol reagent for 1 h. (b) Magnified view of Figure S1a. Figure S2: (a) Measured absorbance curves of the DAS array (black) before and (red) after drying a 10 μL drop of an aqueous solution of exosome. (b) Table of LSPR peak shifts of the DAS arrays, measured after dipping in pH buffer solutions with pH 7.4, 5.0, and 4.0 for 18 hours.
