# Supplementary material for: Active Accumulation of Spherical Analytes on Plasmonic Hot Spots of Double-Bent Au Strip Arrays by Multiple Dip-Coating
Source: Nanomaterials (Basel). 2019 Apr 26;9(5):660. doi: 10.3390/nano9050660 (PMC6567194; doi:10.3390/nano9050660)
Supplement: Supplementary file 1 [file nanomaterials-09-00660-s001.pdf]

Supplementary Materials

Active Accumulation of Spherical Analytes on Plasmonic Hot Spots of Double-Bent Au Strip Arrays by Multiple Dip-Coating

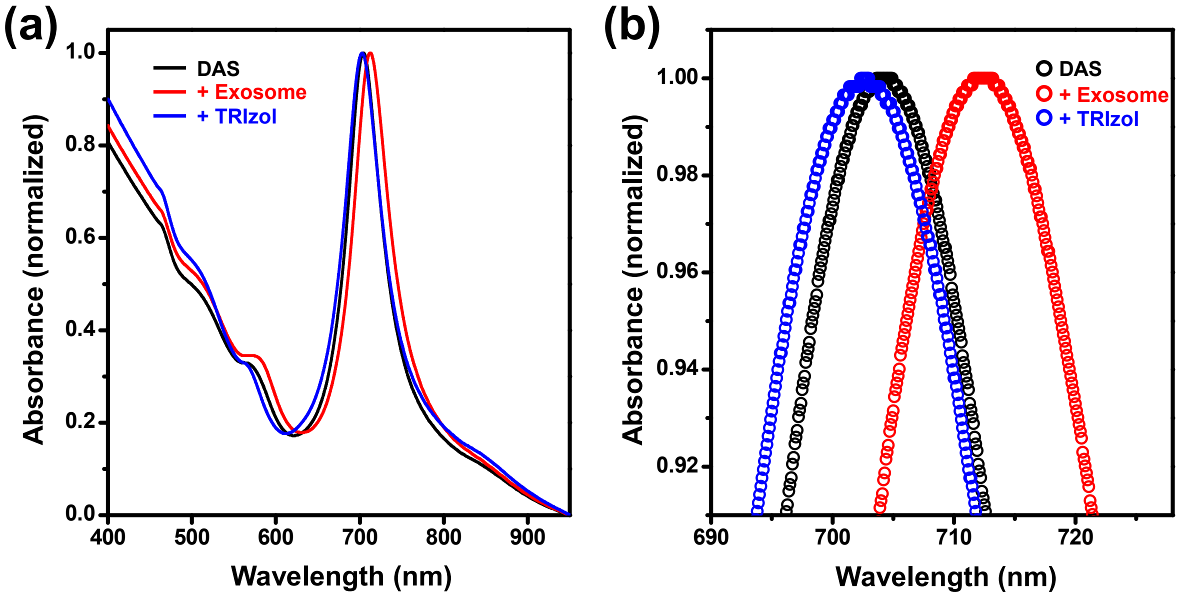

Figure S1. (**a**) Measured absorbance curves of the DAS array (black) before and (red) after dip-coating thrice with exosomes and (blue) after immersion in a TRIzol reagent for 1 h. (**b**) Magnified view of Figure S1a.

(a)

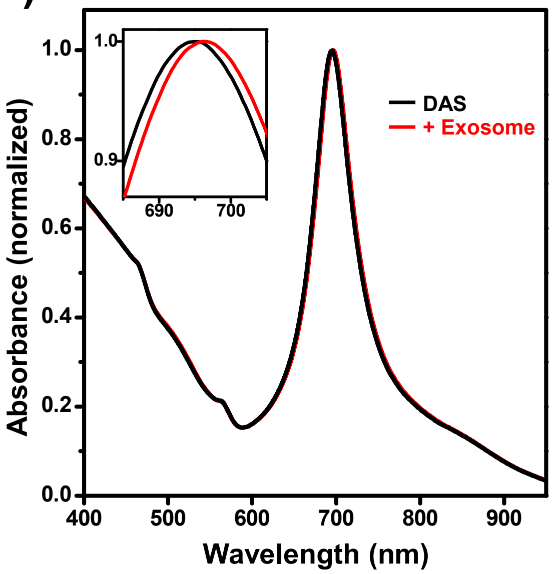

(b)

	Peak shift for 18 hours
pH 7.4	0.2 ± 0.3 nm
pH 5.0	− 0.2 ± 0.4 nm
pH 4.0	− 0.1 ± 0.2 nm

Figure S2. (**a**) Measured absorbance curves of the DAS array (black) before and (red) after drying a 10 μL drop of an aqueous solution of exosome. (**b**) Table of LSPR peak shifts of the DAS arrays, measured after dipping in pH buffer solutions with pH 7.4, 5.0, and 4.0 for 18 h. To confirm the stability of the DAS arrays in the pH buffer solutions, the measurements were done without a dip coating of exosomes or polystyrene beads.
